# A Pilot Multisensory Approach for Emotional Eating: Pivoting from Virtual Reality to a 2-D Telemedicine Intervention during the COVID-19 Pandemic

**DOI:** 10.3390/jcm12237402

**Published:** 2023-11-29

**Authors:** Clelia Malighetti, Ciara Kelly Schnitzer, Sophie Lou YorkWilliams, Luca Bernardelli, Cristin D. Runfola, Giuseppe Riva, Debra L. Safer

**Affiliations:** 1 Dipartimento di Psicologia, Università Cattolica del Sacro Cuore di Milano, Largo Gemelli, 20123 Milan, Italy; 2 PGSP-Stanford PsyD Consortium, 1791 Arastradero Rd, Palo Alto, CA 94304, USA; ciaraks@stanford.edu; 3 Department of Psychiatry and Behavioral Sciences, Stanford University, 401 Quarry Rd, Stanford, CA 94305, USA; syorkwilliams@stanford.edu (S.L.Y.); crunfola@stanford.edu (C.D.R.); dlsafer@stanford.edu (D.L.S.); 4 BECOME Srl, 20133 Milan, Italy; luca.bernardelli@become-hub.com; 5 Applied Technology for Neuropsychology Lab, IRCCS Istituto Auxologico Italiano, 20145 Milan, Italy; giuseppe.riva@unicatt.it; 6 Humane Technology Lab, Università Cattolica del Sacro Cuore, 20123 Milan, Italy

**Keywords:** emotional eating, eating disorder, binge-eating disorder, bulimia nervosa, virtual reality, treatment, somatic marker

## Abstract

Background and Objectives: Emotional eating (EE), or eating in response to negative emotions or stress, can be understood as a manifestation of difficulties regulating emotions among individuals with eating disorders. To date, many virtual reality treatments for eating disorders have focused on body image or exposure methods and have not exclusively targeted EE. There has been a call made by experts in the field for a “new generation” of virtual reality interventions, capable of utilizing virtual reality’s potential more fully. We developed a novel emotion regulation (ER) intervention based upon virtual reality to improve EE among adults with an eating disorder diagnosis. The study hypothesized that a novel ER protocol utilizing evidence-based strategies, as well as innovative techniques, would be feasible and acceptable and show preliminary signals of effectiveness for EE. Materials and Methods: Due to COVID-19, the study pivoted from the original completely immersive intervention to a 2-D intervention deliverable over telehealth. Twenty-one patients were recruited from the Adult Eating Disorders Program within Stanford University to receive seven weekly one-hour virtual experiences (VEs) focusing on ER. Participants were not randomized but, as part of a pragmatic study design, chose between the novel VE-Emotion Regulation (VE-ER) intervention or continuing their treatment as usual. Before and after the seven sessions, participants completed an assessment by filling out online questionnaires. Results: Overall, VE-ER treatment was feasible, and the participant and therapist acceptability of VE-ER treatment was fairly high. In terms of preliminary effectiveness, the results showed a significant reduction in the frequencies of disordered eating behaviors in both groups, but a greater improvement in EE in the VE-ER group and a significant reduction in emotion dysregulation after the treatment. Conclusions: This novel pilot study makes a valuable contribution to the scant literature by demonstrating the feasibility, acceptability, and preliminary effectiveness of combining somatic, multisensory, and cognitive manipulations delivered via telemedicine to help patients with EE to manage their emotions. The findings can serve as the basis for larger, controlled studies evaluating the translation of the somatic marker theory from the research literature into real-world U.S. clinic settings.

## 1. Introduction

Emotion regulation (ER) is a complex process defined as the initiation, maintenance, and modification of the occurrence, intensity, and duration of feeling states [1]. Research over the last decade shows that difficulty with ER is a core transdiagnostic construct across both the eating disorder spectrum and other disorders, such as depression, anxiety, substance use, and borderline personality, thus partly explaining their high comorbidity with eating disorders [2]. Emotional eating (EE), or eating in response to negative emotions or stress, is one of the key manifestations of difficulties with ER within eating disorders [3]. EE is positively associated with both the presence and severity of binge eating [4], but despite its prevalence (20.5%) [5] and important transdiagnostic features, it has not received much research attention.

EE, in particular, was found to increase in the context of psychological distress during the COVID-19 lockdown and isolation [6], with EE predicted by higher depression and anxiety levels [7]. These findings support prior research [8,9,10], which shows a relationship between EE and stress, modulated by the body mass index (BMI) [11,12,13]. Indeed, EE has been associated with emotion regulation difficulties [8,9] and a higher level of alexithymia (difficulty experiencing, identifying, and expressing emotions) [14]. Some posit that higher alexithymia impairs one’s ability to identify emotional states and to distinguish them from internal signals of hunger and satiety, resulting in food consumption as a way to regulate emotions [14,15], with downstream effects on BMI [15,16,17]. Research supports a relationship between EE with higher weight and challenges with weight [9,18,19]. Given the presence of weight stigma and weight-based discrimination in society [20], these weight changes may evoke significant distress and result in a vicious cycle between negative moods and eating.

Given the links between emotion dysregulation and EE [21], existing interventions targeting EE have utilized mindfulness and emotion regulation skills training. Mindfulness can be described as a state of purposeful attention towards a momentary experience, holding an open, accepting, and non-judging attitude [22]. At a neurobiological level, this type of mental training may have an effect on the plasticity of the brain structure and functioning, such as attention control, emotion regulation, and self-awareness [23]. Several studies have shown mindfulness techniques improve mental health among clinical populations. A systematic review and meta-analysis of mindfulness-based interventions for binge eating specifically found mindfulness training to reduce binge eating severity at the end of treatment and to reduce depression and improve ER; however, benefits were not maintained at follow-up [24]. Mindfulness-based approaches can also address issues specific to emotional eating through the modification of ER. Mindfulness techniques are related to healthier eating, facilitate the acceptance and tolerance of negative emotions, and exert their beneficial effects on a range of outcomes through attention regulation, emotion regulation (including reappraisal, exposure, extinction, and reconsolidation), and enhanced executive functioning, most notably in terms of inhibitory control [22].

Despite the fact that such interventions that teach mindfulness and/or other emotion regulation skills have shown promise in reducing symptoms [22,25,26], innovative technologies and theoretical approaches offer the promise of improved outcomes and reduced attrition.

Virtual reality has been studied as a successful technique for enhancing psychological treatment [27]. Virtual interventions allow researchers and clinicians to recreate life-like experiences in a safe environment and, in particular, have been widely used in populations [28]. Further, virtual reality enhanced treatments for eating disorders show superior efficacy, faster results, and better maintenance compared to non-virtual reality delivery [29].

Previous authors indicated that virtual experiences integrated with biofeedback, mindfulness, and imagery techniques offer a therapeutic approach that helps patients make new associations with negative memories, thereby altering emotional states [30]. This is typically performed through cognitive techniques; for example, a patient might be encouraged to envision a future outcome turning out positively [31,32]. A meta-analysis that investigated imagery rescripting as a clinical intervention for aversive memories found it to be effective for reducing psychological symptoms [33]. An immersive intervention can modify the user’s ER by allowing for the modification of the emotional response across a reappraisal of emotional stimuli and therefore inspiring new actions. This re-evaluated experience is more easily encoded into memory. In this light, immersive intervention integrated with imagery rescripting may be able to modify emotional core beliefs [34].

A recent paradigm of embodied cognition reconceptualized the nature of cognition by specifying that cognition occurs in a specific bodily environment [35]. In other words, the mind must be considered and studied in relation to a physical body that interacts with the world. Hence, human cognition is not centralized, abstract, and clearly distinct from the peripheral modules of input and output, but has deep roots in sensorimotor processing [35].

Recently, it has been suggested that our brain generates simulations that filter the incoming sensory input, guide action, and cause perception and other psychological phenomena, including emotion [36,37,38].

Barret explains the following: “Our brain constructs meaning by correctly anticipating (predicting and adjusting to) incoming sensations. Sensations are categorized so that they are: (i) actionable in a situated way; and therefore (ii) meaningful, based on past experience. When past experiences of emotion (e.g., happiness) are used to categorize the predicted sensory array and guide action, then one experiences or perceives that emotion (happiness)” [37]. In this light, we must alter the body’s experience related to an emotion in order to regulate that emotion.

Relatedly, Damasio, in his somatic marker theory [39], posits that emotions are changes in both our body and brain states. Over time, emotions and their corresponding bodily changes, or “somatic markers”, become associated with particular situations and their past outcomes. Once formed, the somatic markers are reactivated every time the person encounters similar situations to those that originally induced the emotion reaction. The reactivation of the somatic markers calls forth the historically associated body state. Somatic markers permit the comparatively fast pre-selection of the relevant alternatives, which are then subjected to more detailed cognitive processing for final decision-making. In this way, somatic markers increase the efficiency and accuracy of human decision making [40]. In other words, when we experience a situation similar to one in the past, our body reproduces the same emotional and behavioral responses it had learned in the previous situation. This automatic mechanism makes it difficult to modify the emotional reaction with a more adaptive one. Rescripting a somatic marker through immersive experiences has the exciting potential for patients to create a new emotional experience with their bodies, which could in turn influence their ER and eating behavior (e.g., EE).

To date, many virtual reality treatments for eating disorders have focused on body image or exposure methods and have not exclusively focused on EE [27]. There has been a call made by experts in the field for a “new generation” of VR interventions, capable of utilizing virtual reality’s potential more fully [27]. To our knowledge, there are no studies that have investigated the combination of virtual reality, mindfulness techniques, and imagery rescripting to treat EE starting from a somatic view of emotional experience. Rescripting somatic markers through immersive experiences involving the body would represent a novel direction in the treatment of EE.

A recent promising case study [41] reported on a patient who decreased EE and improved ER after receiving a novel ER-based virtual reality intervention that used rescripting techniques based on Damasio’s somatic marker theory [39]. This led us to undertake the current, larger study to further investigate the acceptability, feasibility, and preliminary signals of effectiveness of this same innovative protocol using virtual reality to improve current EE among adults with a history of an eating disorder diagnosis. Given links between emotion dysregulation and EE [21], the intervention utilized evidence-based strategies, such as mindfulness and imagery rescripting, as described below. However, due to the timing of the COVID-19 pandemic requiring lockdown, during which all clinicians were required to work remotely, the virtual immersive scenarios could not be delivered using a 3-D head-set virtually as originally planned (as it would have required in-person sessions to access the head-set). To enable access to the intervention during this period, the study pivoted from the originally planned immersive virtual reality delivery to 2-D video conferencing, which enabled the treatment to be delivered remotely. Previous studies [42,43,44,45] have found 2-D virtual experiences (VE) to be effective in improving the overall psychological and emotional well-being of adults during the COVID-19 pandemic.

We hypothesized that a novel ER protocol utilizing evidence-based strategies, such as mindfulness and imagery rescripting, as well as innovative techniques, such as virtual experiences, would be feasible and acceptable and show preliminary signals of effectiveness for EE among patients with a history of prior eating disorder diagnoses.

## 2. Materials and Methods

### 2.1. Participants

Twenty-one participants were recruited between September 2020 and September 2021 from the Adult Eating Disorders Program (AEDP) within Stanford University School of Medicine. All participants were informed about the purpose of the research and provided written informed consent. The study was approved by the Institutional Review Board (IRB) of Stanford University. Inclusion criteria included English speaking adults with a history of a DSM-5 eating disorder diagnosis (e.g., binge-eating disorder, bulimia nervosa, other specified feeding and eating disorders) from an AEDP clinician and current (i.e., within past month) subjective emotional eating (see Measures, below). Exclusions were current significant suicidal ideation, psychosis or bipolar I (unless stably remitted on maintenance therapy for at least 1 year), and/or any newly initiated (e.g., <1 month) psychotropic medication or adjunctive psychotherapy.

### 2.2. Study Design

Patients were recruited through flyers emailed to all clinicians working in the AEDP. A study team member also informed all clinicians about the study and study eligibility at the regular weekly eating disorder treatment team meeting. Clinicians provided their patients with information about the study. If patients had an initial interest in the study, clinicians could refer their patients to the study by emailing the research coordinator and completing a brief HIPAA-compliant online referral form inquiring about study eligibility. The research coordinator confirmed eligibility by reviewing these forms. Prospective patients were given informed consent and signed written consent as desired. Consent guaranteed the protection of privacy and outlined the risks and benefits, as well as patient rights, with regards to the virtual intervention.

As was considered appropriate and pragmatic for a real-world clinic with paying patients, participants were given the choice of two treatment options: (1) to continue their current clinic treatment as usual (TAU), or (2) to engage in the novel virtual experience for emotion regulation (VE-ER) intervention. Participants had the option to select whichever treatment option best suited their needs and comfort level. As such, eligible participants did not undergo true randomization.

### 2.3. Treatment as Usual (TAU)

Participants that chose TAU continued to receive the evidence-based treatment that they were currently receiving in the Stanford Eating Disorders Clinic [e.g., group or individual cognitive behavioral therapy (CBT), dialectical behavior therapy (DBT), or interpersonal psychotherapy (IPT)]. Participants were asked to complete study assessments/surveys before and after seven weeks.

### 2.4. Non-Immersive Multisensory Virtual Experience for Emotional Regulation Intervention (VE-ER)

The intervention was developed as a collaboration between Stanford’s Virtual Reality Lab Immersive Technology (VR-IT) Clinic, a VR/AR Tech Company, Become-hub, and the Department of General Psychology at the Catholic University of the Sacred Heart. Participants in the VE-ER group participated in a total of seven sessions. The first session included an initial assessment to allow for the greater personalization of treatment. Sessions two and three were dedicated to the emotion regulation component, which aimed to increase the participant’s ability to recognize emotional states. Sessions four, five, six, and seven included an emotional rescripting component that aimed to increase the participant’s confidence in their ability to experience and skillfully manage challenging emotional states. Each session also included an experiential component based on the somatic marker theory (e.g., the anchoring hand gesture) that enables interaction between the narrative and bodily experience. Sessions were 50–60 min. Given space constraints, a further description of the virtual scenarios is in Supplementary Tables S1 and S2 and greater detail on the protocol is in Supplementary Table S3.

Over the course of seven weeks, participants were guided through a series of scenarios, each connected to the development of skills for improved ER and eating. Before every session, the participants were sent the Zoom link. The first session assessed the participant’s symptoms of EE and disordered eating patterns in detail to personalize treatment, including obtaining the frequency of disordered behaviors (large binges, small binges, purges, EE) All subsequent sessions began with a brief assessment of the frequency of disordered eating over the previous week (e.g., large binges, small binges, purges, EE), which was documented in the therapist’s session note. Starting with session 3, this was followed by a review of the homework from the previous session. The goal of the two ER sessions was to help participants improve ER abilities and to increase their awareness of emotional states. The first experience was a therapist-guided mindfulness breath and bodily sensation exercise (focus on attention).

This was followed by introduction to the virtual scenario in which the patient was guided to implement mindfulness-based strategies to explore landscapes while identifying internal states (e.g., the narrator within the scenario guided patients to attend to their bodily sensations, the movement of their breath in and out, colors, and images, etc.).

Afterwards, the therapist asked the patient to recall the immersive experience and identify a moment that they experienced pleasant emotions. This was referred to as a “safe place” that the therapist helped the patient to “anchor” by linking the positive emotions experienced during the virtual environment to an actual positive experience the patient had in the “real world”. While doing so, the therapist encouraged them to generate vivid details about the real-life experience while making a hand gesture (closing their thumb between their four fingers) in order to anchor this positive feeling within the body.

Participants were taught strategies for accessing and using safe place visualization and somatic markers (i.e., the hand gesture that creates an anchor by linking the positive emotion experienced in the virtual environment to an actual experience the patient had in the “real world”. The greater the detail, the more vivid the recollection and the subsequent re-experiencing to regulate the effect at appropriate times during the week. For daily homework, the participant practiced re-experiencing the immersive moment by listening to an audio or video file of the session. See Supplementary Tables S1–S3 for additional detail.

The emotional rescripting experiences represent a metaphorical journey towards healing (i.e., beginning in a fragile state, overcoming obstacles, and practicing skills). Each scenario has a specific meaning and associated obstacle (see Supplementary Table S3). Emotional rescripting sessions began with the same five-minute mindfulness exercise described above (“focus on attention” exercise), followed by the immersive experience. During each metaphorical experience, the participant made use of real-life objects or sensorimotor reinforcements, with the aim of encouraging changes in the somatic markers and increasing the body’s involvement in the immersive experience. After the video was viewed, the patient was asked to identify their emotions and localize them in their body. If the emotions were predominantly positive, the therapist amplified the emotion by facilitating mindful awareness of the emotion and anchoring it to a real-life experience. If the emotions were predominantly negative, the therapist could help the participant reduce the negative experience by rescripting the negative memories associated with similar emotions and/or through awareness, acceptance, and physicalizing exercises (e.g., treating unwanted content as an object that the participant could visualize as moving into the horizon until it disappeared and/or was contained).

For daily homework, the participant listened to the session while holding a real-life object associated with the scene/emotion. Overall, the participant was rehearsing and reinforcing stability and self-confidence at each stage of the journey.

### 2.5. Measures

Data were collected remotely through the use of Qualtrics, a secure online survey tool. Descriptive data were collected during the first session. In both conditions (VE-ER and TAU), preliminary signals of effectiveness data were collected before the intervention (at baseline) and after seven sessions (the duration of the VE-ER condition).

#### 2.5.1. Descriptive Variables

A demographic form inquiring about age, sex, race/ethnicity, socioeconomic status, and concurrent treatment was obtained at baseline.

#### 2.5.2. Feasibility

Outcomes assessing feasibility included the following: (1) number of therapists who, after attending an information session about virtual reality and eating disorders, chose to attend the two study training workshops; (2) number of providers who, after attending the training workshops, subsequently enrolled as study therapists; (3) number of eligible (clinician-referred) patients who chose to enroll in the study; (4) number of sessions attended and number of study completers. The choice of these definitions of feasibility were based on the research literature [46].

#### 2.5.3. Acceptability

The Client Satisfaction Questionnaire-Revised (CSQ-R) [47] was used as the acceptability measure. This eight-item measure is used to assess satisfaction with a product or service and was modified to be suitable for therapists and participants in both the VE-ER and TAU conditions. Scores based on the CSQ-R range from 8 to 32, with higher scores indicating greater satisfaction.

#### 2.5.4. Exploratory Outcomes of Effectiveness

Before and after the intervention (at baseline and after seven sessions) participants from both conditions (VE-ER and TAU) were asked to complete the following measures.

- Difficulties in Emotion Regulation (DERS) [48]. This self-report scale asks respondents to rate how they manage their emotions on a 5-point Likert scale ranging from never to always. Six subscales emerge from the questionnaires: (1) “The inability to accept emotional responses”, (2) “Impulse control difficulties”, (3) “Difficulty engaging in goal-directed behavior”, (4) “A lack of emotional awareness”, (5) “Lack of emotional clarity”, and (6) “Limited access to emotion regulation strategies”. Higher scores indicate greater problems with emotion regulation. In this study, we considered only the Total Scores that ranged from 36 to 180. There are no standardized clinical cutoffs for this measure; however, prior research suggests that the clinical range based on the DERS total score varies from averages of approximately 80 to 127 [49]. This measure has good internal consistency, good test-retest reliability, and predictive validity [50].
- The Dutch Eating Behavior Questionnaire (DEBQ) [51]. The DEBQ is a 33-item self-report questionnaire that assesses three distinct eating behaviors in adults: (1) emotional eating, (2) external eating, and (3) restrained eating. Items on the DEBQ range from 1 (never) to 5 (very often), with higher scores indicating greater endorsement of the eating behavior. Similar to the DERS, there are no standardized clinical cutoffs. Research community samples suggest a score > 3.25 as the 80% percentile. The DEBQ’s subscales have good internal consistency, test-retest reliability over a 4-week period, and criterion validity [51]. For reliability, De Cavalho and colleagues identified an acceptable Spearman correlation coefficient (rho  >  0.30 and *p*  <  0.05) and Cronbach’s alpha (*α*  ≥  0.70) for all DEBQ items of the online format [52].
- Frequency of disordered eating. At the beginning of each session in both conditions, therapists assessed the participant’s frequency of disordered eating. This information was entered into a Therapist Note on Qualtrics. Preliminary signals of effectiveness were determined by changes in the frequency of disordered eating behaviors over the previous 7 days (e.g., number of EE episodes, evaluation of the trend of EE, number of objective binge episodes (OBEs), subjective binge episodes (SBEs), purging episodes). EE episode frequency was assessed using a 5-point Likert scale: Never (1), Seldom (2), Sometimes (3), Often (4), Always (5). Binge episodes were distinguished as objective or subjective as defined by the Eating Disorder Examination Questionnaire (EDE-Q). OBE and SBE episodes were assessed asking for a specific number of episodes over the prior week.
- The Acceptance and Action Questionnaire (AAQ-II) [53]. This seven-item measure assesses psychological inflexibility and experiential avoidance. Items range from 1 (never true) to 7 (always true). The scale is scored by summing the seven items. Higher total scores indicate less flexibility, while lower total scores mean more flexibility (total range: 7–49). This measure has good internal consistency (α = 0.88) and good test retest reliability over 3 and 12 months at 0.81 and 0.79, respectively.
- Weight Efficacy Life-Style Questionnaire (WELSQ) [54]. The Weight Efficacy Lifestyle Questionnaire (WELSQ) is a commonly used measure of eating self-efficacy consisting of 20 items and five situational factors (negative emotions, availability, social pressure, physical discomfort, positive activities). Respondents rate their confidence to resist eating in certain situations on a 10-point Likert scale ranging from 0 (not confident) to 9 (very confident). The WELSQ yields five subscale scores ranging from 0 to 36. High WELSQ scores indicate a higher self-efficacy to resist eating.

### 2.6. Data Analysis

Descriptive statistics (e.g., mean, standard deviation) were used given the small sample size of the study. Exploratory analyses using the Mann–Whitney test were performed to analyze differences between groups based on pre- and post-intervention data. The Wilcoxon test was used to evaluate the change within the two groups. Unfortunately, in the virtual group, only 5 out of 10 received the post treatment questionnaires. This was due to personnel error associated with staffing changes during COVID-19. In addition, there were no differences in the VE-ER group based on baseline measures between the five participants who received post-treatment questionnaires versus the five who did not, including no differences in binge eating severity, measures of ER, and EE. Given the substantial missing data in the VE-ER group, the decision was made to analyze paired *t*-test within-group differences based on the exploratory measures and not to compare the two conditions using independent t-tests to assess between-group differences. Participants’ frequency of disordered eating was obtained from the therapist note (with complete data for all participants); a repeated measures ANOVA was carried out for pre-post treatment evaluation between conditions. We adopted a significant *p*-value as <0.05. The Statistical Package for Social Sciences (SPSS, version 27) was used for all analyses.

## 3. Results

### 3.1. Descriptive Analysis

Participants were, on average, 41.7 (SD = 15.7) years of age, female (90.5%), Caucasian (81%), at least college educated (100%), had a history of a binge eating disorder (BED), and had previous experience with psychotherapy (100%). Nearly half of them were engaged in other treatment for a psychiatric disorder (42.9%), the majority (90%) for medication management.

### 3.2. Feasibility

#### 3.2.1. Therapists

Eleven therapists attended an initial information session about virtual reality and eating disorders. Of these 11, 10 enrolled as study therapists. All therapists were eating disorder specialists at the doctoral level (PhD, PsyD, MD). All were female.

#### 3.2.2. Patients

Twenty-three patients were referred by clinic providers to the study. Eleven patients expressed interest in the VE-ER treatment and twelve expressed interests in the TAU condition. Of the 11 eligible participants that expressed interest for the VE-ER condition, 10 chose to enroll, and of the 12 eligible participants for TAU condition, 11 chose to enroll. There was a 100% completion rate in both conditions. The average number of sessions attended was 7 out of 7 for both groups. The majority (90%) of the 10 participants in the VE-ER condition who received all surveys completed surveys; however, as per above, not all participants in this condition received all of the post-treatment assessments.

### 3.3. Acceptability

#### 3.3.1. VE-ER Intervention

**Figure 1:**
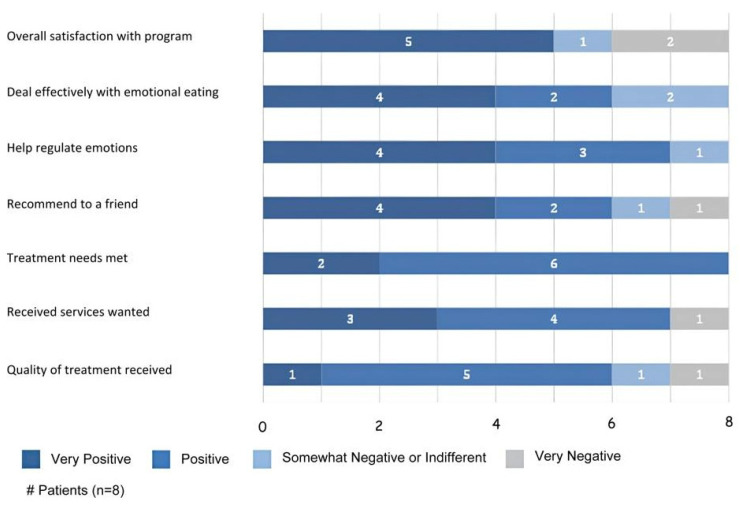
Patient post-treatment satisfaction CSQ-R scores.

The mean client satisfaction questionnaire-revised (CSQ-R-Patients) score was 24.75 (SD = 6.7, *n* = 8) out of a possible 32, indicating fairly good treatment satisfaction (Figure 1). The majority of the participants, 75% (6/8), rated the quality of the treatment as “excellent” or “good”. Generally, two participants (25%) had neutral-to-negative feedback about the treatment. Six of the participants (75%) reported that they would participate in the intervention again if they sought services for emotional regulation. Five of the participants (62.5%) reported that they would participate in the intervention again if they sought services for emotional eating.

Participants were also asked about their satisfaction with the 2-D virtual scenarios and technology. All but one participant (87.5%, 7/8) reported that the journeys clearly conveyed the various emotional themes; one (12.5%) found them to be only “a little” clear. When asked to rate their satisfaction with the narrator’s voice, participants responded as follows: 12.5% (1/8) “very satisfied”, 12.5% (1/8) “mostly satisfied”, 50% (4/8) “indifferent or mildly satisfied”, or 12.5% (1/8) “quite dissatisfied”. Participants rated the sound effects as helping “a great deal” (25%; 2/8), “somewhat” (37.5%, 3/8), or “didn’t really help” (37.5%, 3/8). Most of the participants found the virtual scenarios to be “realistic” (50%, 4/8) or “a little realistic” (25%, 2/8); two found them to b “not at all” realistic (25%). Open ended -responses from these two participants suggest that they did not find them to be realistic because of connectivity issues (“choppy movement” during the video) or the stimuli not being relatable to real-world stimuli. One participant wrote “It felt like I was watching a cartoon and the exposures were not advanced enough cinematically without a virtual reality setting. This really needs to be in a virtual reality setting—I understand covid upended the in-person study, but the team needs to explore an at home virtual reality experience…”. All but one participant (87.5%) reported being at least a little successful at using the audio recordings outside of treatment sessions.

In an open-ended response format, all participants were asked about the most helpful aspects of the intervention. Fifty percent of participants (4/8) stated that talking with the therapist was helpful; many felt that a strong therapeutic alliance was critical, in addition to meaningful reflection, processing, and insights following immersive scenes. Some responses additionally added that “the physical participation of holding a compass, or carrying a backpack” and “accessibility to videos as needed” were helpful. Participants were asked about the least helpful aspects of the intervention. Thirty-eight percent of participants (3/8) responded that audio or video quality was the least helpful; participants found the video to be “choppy” or “botched” intermittently during screenshare. One participant commented, “The program (or maybe the therapist?) did not do a good job relating the videos and topics on emotion regulation to binge eating”.

Participants were asked how they would improve the intervention. Twenty-five percent of participants (2/8) reported that they would improve the quality of the VR-ER videos; for example, one participant stated, “I had hoped the immersive experiences would have felt more realistic”. Other responses included using 3D virtual reality (37.5%, 3/8), personalizing videos for each participant (25%, 2/8), being more open to the intervention (12.5%, 1/8), and having videos be more easily accessible for between-session review (i.e., not emailed but accessible on an app) (12.5%, 1/8).

#### 3.3.2. TAU Treatment

The mean CSQ-R score was 24.50 (SD = 1.58) out of possible 32, indicating good treatment satisfaction. The majority of the participants, 80% (8/10), rated the quality of the treatment as “excellent”, and 20% (2/10) rated the quality of the treatment as “good”. Overall, 90% (9/10) of patients were satisfied with the TAU treatment, and 10% (1/10) of patients were indifferent or mildly dissatisfied.

#### 3.3.3. Therapists’ Satisfaction with Virtual Intervention

**Figure 2:**
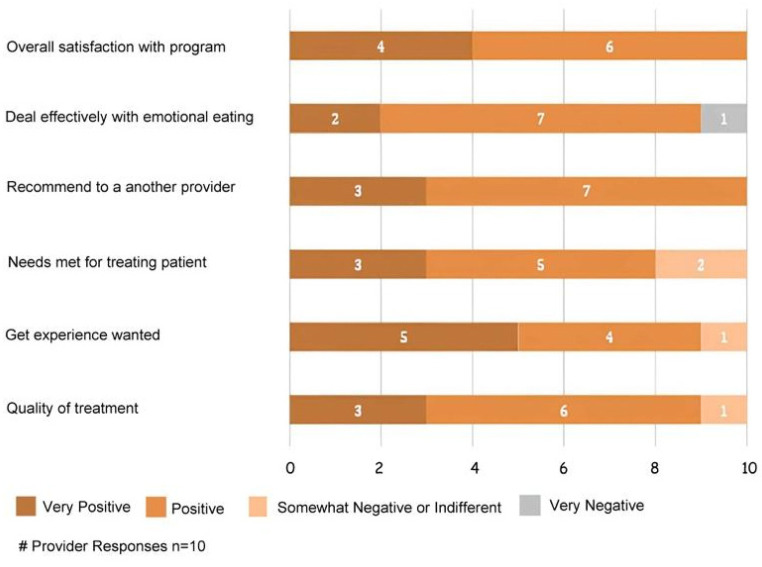
Provider post-treatment satisfaction CSQ-R scores.

The therapist’s mean CSQ-R score was 22.71 (SD = 1.89, *n* = 10) out of a possible 32, indicating overall fairly good satisfaction in delivering the intervention (Figure 2). Ninety percent of therapists (9/10) rated the quality of the intervention as either “excellent” (3/10) or “good” (6/10) with one therapist (10%) rating it as “fair”. This same therapist rated the intervention as making “no difference” in the participants’ EE. Most therapists, 90% (9/10), thought that they would use the intervention again in the treatment of eating disorders.

Therapists were also asked specifically about the manual and homework. Seventy percent (7/10) of therapists found the manual to be “clear”, whereas 30% (3/10) found it to be “a little” clear. Most therapists (60%, 6/10) felt “comfortable” or “very comfortable” administering the intervention, whereas 40% felt “a little” uncomfortable (4/10). All therapists (100%) felt “successful” or “very successful” at using the videos for the immersive intervention.

Therapists were asked about the most helpful aspects of the intervention and responded in an open-ended format. Eighty percent of therapists (8/10) reported that the metaphorical journeys were helpful and that the post-processing and discussion that occurred after video review were particularly impactful. Therapists described using cognitive strategies, including cognitive restructuring, reframing, and developing coping phrases, to build motivation, confidence, and resiliency; to target cognitive rigidity; and to rescript unhelpful narratives. One therapist stated, “the metaphors in the re-scripting videos were… effective in helping to change rigidity related to expectations she had for herself, and empowering her with the sense that she has internal resources she can access at any time to overcome challenges”. Another therapist commented, “The metaphorical journeys… seemed to help reinvigorate her to commit to treatment, as motivation was somewhat low when initially referred to therapy. The visual aspect of therapy also seemed to help keep her engaged as she reported difficulty with engagement in the DBT group and her existing [individual] therapy. The videos also helped patients manage urges to sit w/cravings”. Some therapists commented that the videos helped to evoke different emotional states enabling either exposure to negative emotions or the promotion of relaxation states. Sixty percent of therapists (6/10) reported that using the hand gesture or a physicalizing exercise was helpful.

Therapists were also asked about the least helpful aspects of the intervention with an open-ended response format. Thirty percent of therapists (3/10) reported issues with video quality or technical problems (e.g., audio delays, issues with screen share). For example, the “patient found it difficult to feel truly ‘immersed’ in the intended emotional experience”. Thirty percent of therapists (3/10) found the hand gesture to be unhelpful. Therapists were asked what changes they recommend for the VE-ER environments. Fifty percent of therapists (5/10) recommended creating a more realistic immersive experience, including utilizing 3-D virtual reality or making refinements to specific immersive environments. Forty percent of therapists (4/10) reported that they would improve the quality of the narrating voice. One therapist responded with, “not necessarily a change but a point to consider-one of the journeys (The Hero and the Dragon) was triggering for this pt who has a trauma history. The journey became both a trauma exposure and an exposure related to emotional eating… As these journeys are visual (and ideally immersive), [it] may be worth adding a disclaimer or disclosing the scenery of the videos ahead of time in order to enhance safety for the pt”.

### 3.4. Exploratory Outcomes of Effectiveness

An independent sample t-test was performed to evaluate differences between the two groups at baseline (VE-ER vs. TAU group). The results showed no significant differences between the two conditions for any of the baseline characteristics (Table 1).

A repeated measures ANOVA was performed to evaluate differences between group frequencies of disordered eating from the therapist note. Results (Table 2) showed a reduction in the frequency of objective binge eating, subjective binge eating, and EE (#OBES, #SBES and #EE) in both groups. Furthermore, results showed an interaction effect of the two conditions and time on EE episodes; specifically, there was greater improvement in the VE-ER group. Results showed no other significant differences between the two groups over time.

Finally, a within-group analysis was performed to evaluate differences between pre- and post-treatment based on measures in both groups. Results (Table 3) showed a significant reduction in emotion dysregulation (DERS_TOT) post-treatment in the VE-ER group (*p* value = 0.42; *Z* value = −2.032) and no other significant changes in the other measures. The TAU group did not show any significant differences from pre- to post-treatment.

## 4. Discussion

The current study is the first to (1) elaborate on a virtual experiential intervention focused on using emotion regulation strategies for emotional eating and binge eating disorders, (2) integrate somatic manipulation and mindfulness exercises with virtual scenarios in the treatment of eating disorders, and (3) perform a pilot test of a virtual experience for emotional eating in a real-world outpatient eating disorders clinic. In a small non-randomized study, we tested the feasibility, acceptability, and preliminary signals of effectiveness of the intervention for improving emotional regulation over eating among adults who had prior eating disorder treatment with a TAU evidence-based eating disorder intervention comparison control. The COVID-19 pandemic prevented the delivery of the virtual immersive scenarios using a 3-D head-set in person. Instead, the scenarios were delivered remotely via 2-D video conferencing software (versions number 5.2.1-5.8.0). Previous studies [36,37,38,39,40] showed the effectiveness of the 2-D experience of virtual scenarios in improving participant’s overall psychological and emotional well-being during the COVID-19 pandemic.

Overall, participant retention for treatment was high, with 100% (10/10) of the VE-ER group and 100% of the TAU group completing the interventions. This low rate of attrition compares quite favorably with other psychotherapy trials for binge-eating disorders, which report drop rates of 4 to 34% [55,56,57,58]. This study’s lower attrition rate may be associated with the ability to increase participants’ motivation for change, a finding consistent with the literature based on extended reality interventions for eating disorders [59].

Furthermore, therapist interest in this technology-assisted intervention was reasonably high, with 54.55% (6/11) of invited therapists joining the study. In general, these data suggest that the adoption of VR-ER appears to be feasible within a real-world clinic, which is an important issue from the perspective of implementation science. This finding confirms other research showing that clinicians have a positive attitude towards technology and increasing interest in learning extended reality [27].

In regards to acceptability, participant and therapist CSQ-R scores for VE-ER treatment were in the moderate range at 24.75 and 22.71, respectively, out of a possible total score of 32. In regards to participant satisfaction, the majority suggested that the treatment helped them to better regulate emotions and emotional eating, with at least half reporting that they would participate in VE-ER again. In regards to the therapist’s satisfaction, the majority of them would recommend the VE-ER to another provider treating emotion dysregulation and considered the intervention “excellent” and “good”.

In addition to the therapeutic alliance and cognitive processing or rescripting that occurred during the sessions, some patients considered the physical participation of holding objects to be among the most helpful aspects of the VE-ER intervention. From the therapists’ view, one of the most important aspects was the opportunity to discuss and elaborate on the metaphorical meaning of the scenarios. These data add to the extensive scientific and clinical literature [60] supporting the importance of a therapeutic alliance for work in the clinical setting. These findings further suggest that the addition of bodily involvement is a potential element of therapeutic change.

However, regarding the technology and the virtual scenarios, patient and therapist feedback included the suggestion to convert the technology to 3-D virtual reality, as well as to improve the narrator’s voice and sound effects. Two participants found the virtual scenarios to be not at all realistic because of the stimuli not being relatable to real-world stimuli. Please see below for additional suggestions for future studies.

Despite these recommended enhancements, our study found signals of VE-ER intervention effectiveness. Notwithstanding the pilot nature of this study, the VE-ER intervention did appear to result in a significantly greater rate of EE reduction compared to TAU by the end of treatment. In other words, while both groups reduced EE, there was a greater improvement in the VE-ER group. In addition, there was a significant reduction in emotion dysregulation (DERS-TOT scores) only for the VE-ER intervention, with post-intervention DERS-TOT scores close to the non-clinical average range [49]. Consistent with these results, in the VE-ER group only, there was a trend for decreased EE based on the DEBQ_E subscale score and an increasing trend in the ability to resist eating while experiencing negative emotions (based on the WELSQ_NE subscale score) from baseline to the end of treatment. These findings suggest the clinical utility of combining somatic, multisensory, and cognitive manipulations for helping patients with EE to manage their emotions. They are intriguing given the lack of significant changes in emotion regulation from pre- to post-treatment in the TAU group that received a gold-standard treatment.

Our preliminary findings about effectiveness support our expectations regarding the utility of combining somatic marker and virtual scenarios based upon narrative, metaphorical experiences in treating emotional eating.

Specifically, the initial virtual scenarios focus on helping patients identify, experience, and accept different emotional states. These scenarios were chosen given evidence that patients with binge eating disorders often have difficulties managing emotions (such as difficulties identifying and classifying the emotional states and/or difficulty in refraining from impulsive behaviors rather than behaving in ways related to their values and long-term goals) [61]. As noted, the somatic marker theory [39] emphasizes that our emotions have a strong somatic component contributing to decision-making. As such, maladaptive emotional coping strategies may be the result of past negative experiences that then become habitual and automatic ways of responding.

Our virtual scenarios offered a multisensory experience that seemed to help patients improve interoceptive awareness, to be connected to and focused on their internal body emotional sensations in a nonjudgmental way. Somatic objects promoted sensory stimulation at a tactile level, while virtual scenarios enabled visual and auditory stimulation such that a multisensory experience was ensured.

The nonjudgmental focus on emotional states could have enabled them to become more accepting of negative emotions, rather than feeling compelled to change them by eating or in other ways that might be harmful. Several previous studies showed the efficacy of VR in treating and or/preventing anxiety and pain in patients with eating disorders [62] and in mental health [63].

Other virtual scenarios focused on emotional rescripting may have offered patients a fresh perspective on events that happened in the past, thereby eliciting new feelings, identifying unmet needs, and breaking negative automatic responses (including thought processes and behaviors) associated with emotions and eating.

Second, the engagement of the body could have enabled the processes mentioned above to be fostered, spurred by the use of somatic objects and the multi-sensoriality of virtual scenarios that allowed patients to be stimulated at a cognitive, tactile, auditory, and visual levels.

Finally, the use of mindfulness techniques combined with the use of metaphors and virtual scenarios may have shown the transformative potential of VR [42,64] in reinforcing emotional resources and abilities.

There were numerous non-significant changes based on the DEBQ subscales (e.g., external eating and restriction), as well as the WELSQ (measuring eating self-efficacy), and AAQ-II (measuring psychological flexibility) scales. Participant scores based on the AAQ-II in both groups remained in a clinical range post-treatment [65]. It is interesting, however, to note, regarding the DEBQ subscales, that we started the treatment with a clinical sample with significant findings above 80% from a community norm and were able to drop below that and both the measures. As for the other measures, we hypothesize that our short-term intervention focused on targeting emotion regulation, may be less focused on affecting change in other constructs, such as overall psychological flexibility, or that such effects would be observable over time (e.g., a 6-month post-intervention). It is also possible that we lacked adequate power to detect significant differences due to our small sample size.

The study has several limitations. First, our primary goal was to investigate feasibility and acceptability. As such, the sample size was small (to conserve limited financial resources), data were primarily descriptive, and between- and within-group analyses were exploratory. These exploratory findings need to be interpreted with caution. Additionally, true randomization was not carried out. As described above, patients were given the choice to participate in the intervention or continue with treatment as usual. This was a pragmatic decision to enable a comparison of the intervention with an evidence-based treatment and control for the passage of time, yet remain suitable for patients paying for care in a real-world clinical setting. Moreover, an additional limitation of the study design is the lack of measurement 1–3 months after the end of the study (follow-up) that could have assessed the long-term effectiveness of the interventions preliminarily.

While no significant baseline differences between the conditions were found in Table 1, it is likely that participants with greater concern over their EE were biased to choose the intervention over TAU. Support for this was found in the VE-ER participants’ lower (though not significantly lower) reported self-efficacy to resist eating triggered by negative emotions compared to the TAU condition. Given the small sample size, exploratory analyses did not include control variables, such as scores based on the WELSQ. Furthermore, the study lacked diversity in relation to gender, ethnicity, and socio-economic status. This lack of diversity limits the generalizability of the results. However, these demographics are fairly reflective of the academic eating disorder clinic’s actual patient population, indicating the need to increase access to care for other populations.

In addition, data were missing for many of the post-treatment assessments due to personnel error associated with staffing changes during COVID-19. Finally, the reality of the COVID-19 pandemic also required pivoting from a 3-D fully immersive version of the intervention to a 2-D version that could be screen-shared via telehealth. This is also a strength of the study in that despite this limitation, the study was found to be acceptable, feasible, and showed preliminary effects.

Future studies should include a larger, randomized sample, and follow-up to evaluate the long-term effect. Additionally, converting content to a 3-D version of the intervention with a virtual reality headset for testing is likely to strengthen user experience and outcomes. Collecting physiological measurements, such as skin conductance or heart rate variability, would provide useful objective data to determine the impact of various treatment components. Furthermore, future studies should consider personalizing videos for each participant in ways that take advantage of virtual reality technology. For example, depending on patient preference, the narrator’s voice could be omitted while maintaining ambient sounds or replaced with the therapist’s voice. Other options for personalization include having a selection of accents, genders, intensities, durations, pitches, and timbres. Using a 3-D virtual reality version would also likely improve access to the scenarios for between-session reviews by providing unlimited access to all videos on demand.

Despite these limitations, we believe that this novel pilot study makes a valuable contribution to the scant literature by demonstrating the clinical utility of combining somatic, multisensory, and cognitive manipulations for helping patients with EE to manage their emotions. Overall, VE-ER treatment was feasible (100% completion), with fairly high ratings of acceptability by both participants and therapists. In addition, the VE-ER intervention generated a promising signal of effectiveness. While disordered eating frequencies were reduced in both conditions, there was a greater improvement in EE within the VE-ER group. In addition, emotion regulation only improved in the VE-ER condition. The findings from this pilot study can serve as the basis for larger, controlled studies evaluating the translation of the somatic marker theory from the research literature into real-world U.S. clinic settings.

## Figures and Tables

**Table 1:** Differences between VE-ER and TAU groups at baseline.

Measures	VE-ER Group *n* = 10	TAU Group *n* = 11	t	Sign
	Mean ± SD	Mean ± SD		
DERS_TOT	109.80 ± 22.90	108.64 ± 22.47	0.117	0.908
DEBQ-R	3.09 ± 0.79	2.78 ± 0.50	1.064	0.301
DEBQ-E	4.12 ± 0.49	3.55 ± 1	1.634	0.119
DEBQ-EXT	3.35 ± 0.88	2.99 ± 0.60	1.093	0.288
AAQ_II	33.40 ± 6.50	30.82 ± 5.98	0.948	0.355
WELSQ_NE	9.90 ± 6.80	15.73 ± 6.21	−2.051	0.054
WELSQ_AV	16.40 ± 8.50	19.18 ± 8.32	−0.757	0.458
WELSQ_SP	16.70 ± 8.23	21.36 ± 6.21	0.351	0.157
WELSQ_PD	17.20 ± 9.36	20.18 ± 6.94	−0.834	0.414
WELSQ_PA	22 ± 5.59	24.45 ± 4.84	−1.092	0.288
#OBES	1.50 ± 1.50	0.73 ± 1.47	0.748	0.984
#SBES	1.70 ± 1.98	3.43 ± 2.57	−1.962	0.951
#PURGES	0.60 ± 1.35	0.64 ± 2.11	−0.047	0.75
#EE	3.40 ± 1.07	2.92 ± 0.90	1.26	0.222

DEBQ-R: restrained eating; DEBQ-E emotional eating; DEBQ-EXT external eating; DERS TOT: difficulties in emotion regulation total score; AAQ_II: the Acceptance and Action questionnaire; WELSQ_NE: the negative emotions-Weight Efficacy Life-Style Questionnaire; WELSQ_AV: the availability subscale; WELSQ_SP: the social pressure subscale; WELSQ_PD: the physical discomfort; WELSQ_PA: the positive activities subscale. #OBEs: number of objectively large binge episodes in the previous week; #SBEs: number of subjectively large binge episodes in the previous week; #PURGEs: numbers of vomiting episodes in the previous week. #EE: emotional eating episodes frequency, never (1), seldom (2), sometimes (3), often (4), always (5).

**Table 2:** Between-group differences (VE-ER vs. TAU) in episodes of binges, subjective binges, purges, and emotional eating.

	VE-ER Group (*n* = 10)		TAU Group (*n* = 11)		Anova				
Measures	Pre	Post	Pre	Post	Time		Group × Time		
	Mean ± SD	Mean ± SD	Mean ± SD	Mean ± SD	F	Sig.	F	Sig.	η^2^
#EE	3.40 ± 1.07	1.90 ± 0.73	2.92 ± 0.90	2 ± 0.60	13.07	0.002 *	4.95	0.038 *	0.39
#SBES	1.70 ± 1.98	0.80 ± 1.13	3.43 ± 2.57	1.42 ± 1.24	9.29	0.007 *	2.69	0.117	0.13
#Purge	0.60 ± 1.35	0 ± 0.00	0.64 ± 2.11	0.45 ± 1.50	3.03	0.098	0.869	0.363	0.32
#OBES	1.50 ± 1.50	0.80 ± 1.13	0.73 ± 1.47	0.55 ± 0.88	7.61	0.012 *	0.005	0.947	0.27

* The mean difference is significant at the 0.05 level. #OBEs: number of objectively large binge episodes in the previous week; #SBEs: number of subjectively large binge episodes in the previous week; #PURGEs: numbers of vomiting episodes in the previous week. #EE: emotional eating episodes frequency, never (1), seldom (2), sometimes (3), often (4), always (5).

**Table 3:** Within-group analysis of VE-ER and TAU conditions.

	VE-ER Group				TAU Group			
	Pre *n* = 10	Post *n* = 5			Pre *n* = 11	Post *n* = 11		
Measures	Mean ± SD	Mean ± SD	z	Sign	Mean ± SD	Mean ± SD	z	Sign
DERS_TOT	109.80 ± 22.90	86 ± 26.46	−2.032	0.042 *	108.64 ± 22.47	102.09 ± 20.62	−1.07	0.284
DEBQ-R	3.09 ± 0.79	2.66 ± 0.95	−0.674	0.500	2.78 ± 0.50	2.76 ± 0.72	0.089	0.929
DEBQ-E	4.12 ± 0.49	3.20 ± 0.67	−1.753	0.060	3.55 ± 1	3.38 ± 0.92	−0.561	0.575
DEBQ-EXT	3.35 ± 0.88	3.10 ± 0.99	−1.604	0.109	2.99 ± 0.60	2.97 ± 0.49	−0.102	0.919
AAQ_II	33.40 ± 6.50	30.20 ± 10.68	−1.095	0.273	30.82 ± 5.98	29.91 ± 7.36	−0.66	0.504
WELSQ_NE	9.90 ± 6.80	18.80 ± 5.63	−1.753	0.070	15.73 ± 6.21	15 ± 5.86	−0.67	0.501
WELSQ_AV	16.40 ± 8.50	20.80 ± 9.49	−944	0.354	19.18 ± 8.32	16.82 ± 8.08	−0.66	−505
WELSQ_SP	16.70 ± 8.23	23.40 ± 7.05	−1.214	0.225	21.36 ± 6.21	19 ± 11.27	−81	0.413
WELSQ_PD	17.20 ± 9.36	23.40 ± 8.26	0.000	1	20.18 ± 6.94	20.18 ± 5.28	−0.35	0.720
WELSQ_PA	22.99 ± 5.59	23.40 ± 7.12	−0.406	0.684	24.45 ± 4.69	22.36 ± 6.57	−0.75	0.449

* The mean difference is significant at the 0.05 level.

## Data Availability

The data presented in this study are available on request from the corresponding authors. The data are not publicly available due to the small sample size and confidentiality concerns.

## Supplementary Materials

The following supporting information can be downloaded at: https://www.mdpi.com/article/10.3390/jcm12237402/s1, Table S1: Virtual scenarios in the VE-ER for Sessions 2–7; Table S2: Summary of VE-ER sessions with the respective theme and somatic object; Table S3: Emotion regulation and rescripting’s sessions.
