# Supplementary material for: A Pilot Multisensory Approach for Emotional Eating: Pivoting from Virtual Reality to a 2-D Telemedicine Intervention during the COVID-19 Pandemic
Source: J Clin Med. 2023 Nov 29;12(23):7402. doi: 10.3390/jcm12237402 (PMC10706878; doi:10.3390/jcm12237402)
Supplement: Supplementary file 1 [file jcm-12-07402-s001.zip › jcm-2719180-supplementary.pdf]

Table S1. Virtual scenarios in the VE-ER for Sessions 2-7

Virtual scenarios*	Description
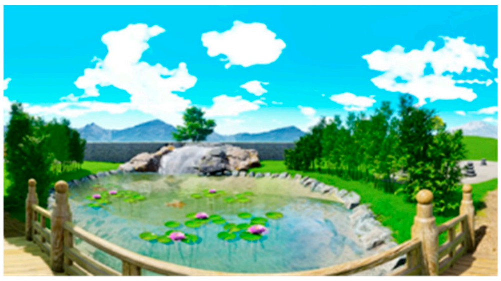	The *Secret Garden* (duration 13:10 minutes) is characterized by a tranquil nature scene intended to help the viewer immediately perceive a sense of calm. Throughout the video the narrative voice guides participants on a walk around a garden on a sunny day.
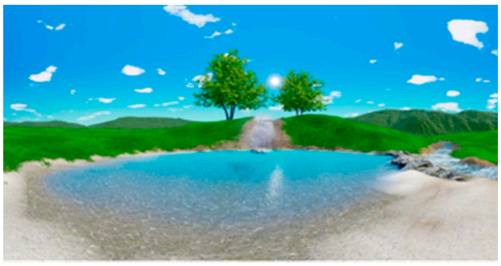	The *Waterfall in the Prairie* (duration 14:13 minutes) scenario takes participants on a walk in a prairie that leads them to a waterfall. As in the first video, this scenario aims to help the participant experience calm and greater awareness of their sensations.
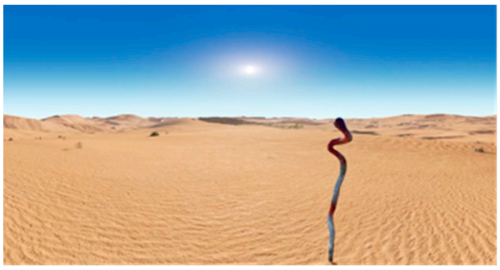	The *Desert and the Oasis* (duration 11:32 minutes) shows a stark and desolate environment. Here participants faced a sandstorm and a scorpion, a symbol of menace, which is overcome by participants in order to return to a more peaceful path. Themes such as loneliness, abandonment, silence, and survival may surface.
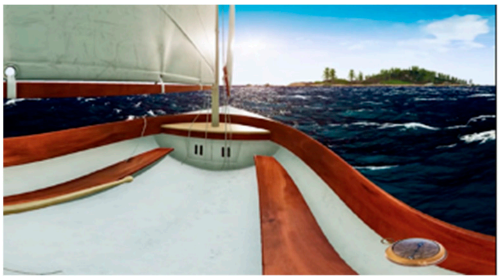	The *Boat and the Sea* (duration 11:12 minutes) involves a journey in a boat, during which a strong storm takes place, perhaps symbolizing a moment of difficulty or anxiety experienced in real life. Participants managed to overcome the storm, and re-experience clear skies and a serene view of the shore.
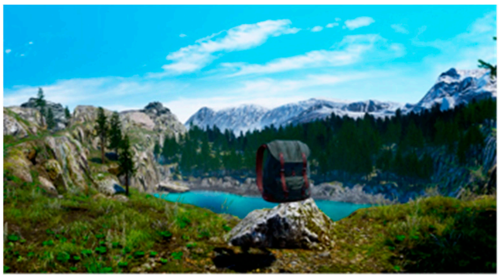	The *Mountain and the Backpack* (duration 13:32 minutes), is a walk along a path in the mountains, during which a heavy stone in a backpack is transformed into a lighter stone, enabling the journey to continue more easily. The scenario involves themes such as challenges, discovery, goals to achieve, resilience, and spirituality.

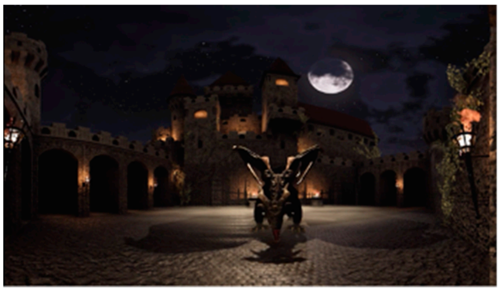	In the final video, *The Hero and the Dragon* (duration 14:05 minutes), participants encounter a frightening dragon that becomes smaller and smaller throughout the video. Participants then manage to reach a door that leads to a treasure chest from which a light emanates, symbolizing the achievement of goals and increasing awareness of their own resources in the face of difficulties.<br>**Images courtesy of Become-hub.*

Table S2. Summary of VE-ER sessions with the respective theme and somatic object

Summary of sessions	Theme
**The Secret Garden**	Build emotional awareness and strengthen mindfulness skills
**The Waterfall and the Prairie**	Build emotional awareness and strengthen mindfulness skills
**The Desert and the Oasis**	Continuing/proceeding despite adversity (sandstorm, scorpion).<br>Somatic marker: a walking stick.
**The Boat and the Sea**	Using a/an (inner) compass to guide you, stay on course (i.e., values-directed action), see the bigger picture instead of overfocus on details, etc.<br>Somatic marker: something that feels like a compass, such as the lid of a bottle or jar.
**The Mountain and the Backpack**	What is keeping the patient weighed down/stuck as they journey through life trying to achieve their goals?<br>Somatic marker: a backpack that can be loaded (to be heavy) and then lightened.
**The Hero and the Dragon**	Patient is the hero of their own quest.<br>Somatic marker: a key

Table S3. Emotion Regulation and Rescripting's sessions

Emotion Regulation Sessions	The role of the experimenter
Introduction to the first two immersive experiences (5 minutes)	Remind patient of the purpose of the emotion regulation intervention and how the intervention will be administered. *"We will now turn to the emotion regulation intervention. As a reminder, the purpose of this intervention is to help you to increase your awareness and ability to recognize your emotional states. I will begin with helping you focus on the present moment and your body. Then you will be immersed in a waterfall landscape and a voice will guide you in the exploration of this scene. The voice will encourage you to pay attention to physical sensations, your breath and to the environment. After the immersive experience we will talk about your feelingss and thoughts about what you experienced. Let me know if you have any questions or need a break."*
Focus on Attention (5 minutes)	*"If you aren't already, please sit in a comfortable position, letting your back adopt a straight but not rigid posture. A dignified and comfortable posture, with the soles of your feet parallel to the ground and your legs uncrossed.*<br>*Place your hands comfortably on your thighs or on your lap. And if you feel comfortable, you can gently close your eyes or keep your eyes open, with a soft, unfocused gaze.*<br>*When you are ready, bring your awareness to the level of your physical sensations, directing your attention to the sensations, pressure and friction in your body at the points where your body is in contact with the chair or whatever is supporting you. Spend a few moments exploring these sensations, in your feet, in your legs, and your hands, back, etc [pause].*<br>*And now direct your attention and focus it on the flow of physical sensations in your abdomen, while the air enters and exits the body [pause]. Try to connect yourself with the*

	flow of the physical sensations in the abdomen for the entire duration of each inhalation and exhalation [pause]. There is no need to try to control your breathing, just let it go [pause]. As you continue to focus awareness on the bodily sensations of the area that enters and exits, sooner or later your mind may move away from the breath, perhaps wandering aimlessly, or be taken over by a thought, a project, a fantasy [pause]. This wandering and being distracted is a normal thing, not a mistake or a failure [pause]. When you notice that your attention is no longer on the breath, pause for a moment to see where the mind has gone, congratulating yourself for being aware of your experience in the moment [pause]. Then, voluntarily turn your attention away from what distracted you and gently bring it back to the sensation of breathing, by focusing completely on breathing in and out. [pause]. Remind yourself from time to time that your only goal is to be aware of your experience moment by moment, the best you can; and that breathing is available to you at all times, when you need an anchor to bring you back to the present moment [pause]. And now, when you're ready, slowly emerge from this experience, redirecting your attention to space and time."<br>*[note: If possible, continue straight from this experience to the immersive environment to avoid moving the focus away from their focus on their bodily experience. If the person appears uncomfortable or voices any concerns, the therapist can feel free to ask about the experience. For example, you can ask: "What was it like to breathe in and out, focus on the breath, have your mind wander, etc?)*
VR Scenarios (12 minutes)	Session 1: The Secret Garden<br><br><u>Introduce Scene: "</u>*We will now start the secret garden immersive experience. In the secret garden experience, we will guide you through a protected place (a large but closed natural garden) where you will find access to a new, internal, world of well-being and awareness (through the portal of the cherry trees).*<br><br>Session 2: The Waterfall and the Prairie<br><br><u>Introduce Scene:</u> *"In addition to the main goal of promoting relaxation, this experience, specifically, will help you lighten your thoughts and emotions providing a safety and protection feeling (an open but safe natural place protected by streams and fences)."*
Identification of the safe place (5 minutes)	After the immersive experience, ask the patient to think through the immersive experience and identify the moment of greatest well-being and serenity. For example:<br><br>"*Go back with your mind to the immersive journey and try to remember the moment when you felt the greatest sense of well-being or serenity. Describe the place and the feeling"*<br><br>If they did not experience a sense of well-being or serenity, therapist can ask what "positive" or "neutral" emotions they felt at any point. Let them describe where they felt them in the scene.
Anchoring the New Somatic Marker (10 minutes)	Create an anchor by linking the positive emotion experienced in the virtual environment to a real experience the patient had in the "real world." Let them close their thumb between their 4 fingers while they are retrieving the real-life experience.<br>*"Now, close your eyes, and --focusing on the emotion that you felt in the VR-- try to recall in your mind an experience that you had in your real life in which you felt this emotion. As you recall this event, close your thumb between the 4 fingers of your hand. Feel the contact of your thumb on your skin and recall as many details as possible. If you feel comfortable, could you share this event with me?"*<br>Let them describe the real-life experience in great detail to you. [The greater the detail, the more vivid the recollection and the subsequent re-experience.]<br>*"Every time that you need to feel this emotion you can make this gesture with your hands . This will bring you back to this present moment and to this positive sensation".*

Homework	Patients at the end of the experience will receive a mp3 or video copy of this immersive experience in order to practice it every day at home before breakfast or in a particular emotional moment of the day.<br>"*I will email you the mp3 and video of this VR experience. Try to listen or watch it before breakfast. This might help you to be more focused as well as more aware of yourself and of what you truly need. If you feel anxious or the urge to eat, try to listen this audio, and make the gesture with your hands that you've already learned. We are trying to help you to be more aware of what is happening in your body and to teach you some skills to face the obstacles that you may meet in your real life.*"

**Emotional Rescripting sessions**	**The role of experimenter**
Check in & review homework (5 minutes)	"How did the homework go last week with listening (or watching) the… scenarios?"<br><u>If completed</u>, process experiences (thoughts and feelings) to facilitate new learning. Use CBT/DBT strategies.<br><u>If not completed</u>, assess barriers to completion and problem solve around overcoming barriers.
Assessment: Therapist Note	Assessment of any changes to emotional eating? (Obtain frequency of behaviors—OBEs, SBEs, and purges, as well as assess presence of emotional eating using the following scale: never, seldom, sometimes, often, always)<br>Any changes to emotional eating compared to last session? (same, worse, better)
Brief Introduction to Rescripting Immersive Experiences	*The somatic marker theory posits that emotions are changes in both our body and brain states. Over time, emotions and their corresponding bodily changes, which are called "somatic markers", become associated with particular situations and their past outcomes. These changes are autonomic and reflect prior experience of that event, usually a negative consequence. Once formed, the somatic markers are reactivated every time the person encounters similar situations to those that originally induced the emotion reaction. The reactivation of the somatic markers reclaims the associated body state. For example, if you were judged in high school for what you ate-- causing understandable negative somatic experiences of anxiety, shame, rapid heartbeat, sweating, etc., then every time you ate in front of those peers, your body may have "memorized" the connection between the negative internal states and eating with others. As such, you might find the same feelings of discomfort present later in life even when those reactions wouldn't make sense, for example, when eating with friends who make you feel safe and have never judged or criticized your eating before.*<br>*An improvement in managing emotions then must depend on a change in the somatic memory of the body. If we experience a negative situation similar to one that we met in the past, our body reproduces the same answers learned in the past (due to body memory). This makes change impossible. It is necessary to rescript this automatic mechanism (somatic marker) to modify the emotional experience. We know that an individual's awareness and understanding of emotions may constitute a necessary step to successfully regulate the emotions.*
Focus on attention exercise (5 minutes).	The same script of previous sessions
VR Scenarios: The Desert and the Oasis Experience; The Mountain and the Backpack; The Boat and the Sea; The Hero and the Dragon	<u>Introduce Scene:</u> *"You will be immersed in a metaphorical journey through ....., holding a .... like object in your hand" [if in person session, can add: "that I will give you at the right time."]. The immersive experience will take 10 minutes. After, we will talk about what you felt and thought during this journey".*<br>During the experience, therapists should observe patients' behavior silently. This observation of the patient's body language can offer useful information. If the patient appears very distressed, for example and the therapists feel the need to intervene, the therapist could stop the video and take a moment to discuss with him/her what they are experiencing and possibly insert a coping skill, reframe, reminder about how repeated exposure can help regulate emotions, etc.)<br><br>As noted, please ask the patient to have a real object similating a … available to grab while they're watching the video. When the narrative voice mentions the …, please remind the patient to grab the real walking stick in order to involve the body for the somatic marker modification. If the patient doesn't have access to a walking stick, the therapist can suggest some alternatives as per above.

Emotion Evaluation (10 minutes)	Purpose: The concretization of what patients felt during the immersive experience will lead them to be able to face and manage their emotions.<br>Once the immersive experience is over, ask the patients to identify and localize in the body what they felt. For example:<br>*"Return with your mind to the immersive experience. How did it feel? Did you feel a negative or a positive emotion?*<br>If they ~~experienced felt~~experienced~~-~~ both positive and negative emotions,~~-~~ always begin with the NEGATIVE. Then, after the desensitization, offer positive reinforcement.<br><br>IF NEGATIVE:<br>Once the emotion is identified, **i~~fn the case of a~~ f negative~~, experience~~** ~~it will be assist the patient in~~ **~~,~~** assist the patient in reduc~~inginged~~ or tolerat~~ing the emotioned~~ing the emotion through the use of awareness and acceptance, physicalizing exercises (treating unwanted content as an object), defusion/desensitization exercises (making it move away to the horizon until it disappears; make it smaller and smaller until it disappears; make it decompose into many small pixels until it disappears), and/or containment<br>Potential questions to ask:<br>*Close your eyes and return with your mind to the immersive experience:*<br>*Q1. (Awareness): Focus on the different parts of your body, WHERE did you feel this emotion?*<br>*Q2. (Physicalizing) Can you give a shape to this emotion? As a natural element, or a Geometric object.*<br>*Q3. What color is emotion?*<br>*Q4. Is the object moving or still?*<br>*Q5.(Systematic desensitization) "Close your eyes and try to focus on the object. It is (color and the name of the object) and it starts to move counterclockwise. As it starts to move, it moves further and further away to the horizon, until it disappears, like a dot."* Or talk about small pixels that crumble and move further and further away as a cloud that branches out and there is no more.<br>If the patient does not manage to make the negative emotion disappear... You can have him/her imagine a drawer or a box where to put the emotion left (containment) or assist them in simply sitting with the emotion (to improve tolerance)<br><br>IF POSITIVE:<br>Once the emotion is identified, **i~~fn case of~~ f positive~~, experience ,~~** ~~it will be~~ **amplif~~y the emotionied~~y the emotion.** *Close your eyes and return with your mind to the immersive experience:*<br>*Q1. Focus on the different parts of your body, WHERE did you feel this emotion?*<br>*Q2. Can you give a shape to this emotion? As a natural element, or a Geometric object.*<br>*Q3. What color is the emotion?*<br>*Q4. Is the object moving or still?*<br>*"Now that you have noticed where you felt this emotion in your body and that you have seen its shape, and its color, try to imagine breathing inside this emotion. Every breath you take, the emotion expands throughout the body."*<br>At this point: **Anchor** by linking the positive emotion experienced in the immersive environment to a real experience lived in the real world. For example: *"Close your eyes and try to recall in your mind an experience that you lived in the real world in which you felt this positive emotion. "Close your thumb between your 4 fingers. Feel the contact with the thumb and try to recall an episode when you felt this same way. Try to retrieve as many details as possible." This positive experience will be memorized in your body and will be available to you every time you need it by making the gesture and using the mp3.*
Homework:	At the end of each session, patients will be asked to repeat the rescripting experience every day before bedtime, using a mp3 audio version of the experience and the real objects associated. The audio version of the intervention will lead the patient to relive the immersive experience by recreating in mind the emotional and somatic screenplay.
